# Assessment of Vascular Remodeling in Coronary Artery Aneurysm and Ectasia Using Optical Coherence Tomography: A Comparative Analysis of Dilated and Non-Dilated Segments

**DOI:** 10.3390/bioengineering13010014

**Published:** 2025-12-25

**Authors:** Patrycja Woźniak, Sylwia Iwańczyk, Konrad Stępień, Maciej Błaszyk, Maciej Lesiak, Weronika Jędraszak, Grzegorz Krupka, Tatiana Mularek-Kubzdela, Aleksander Araszkiewicz

**Affiliations:** 1 1st Department of Cardiology, Poznan University of Medical Sciences, Dluga 1/2 Street, 61-848 Poznan, Poland; 2 Department of Coronary Artery Disease and Heart Failure, St. John Paul II Hospital, 31-202 Krakow, Poland; 3 Department of Thromboembolic Disorders, Institute of Cardiology, Jagiellonian University Medical College, 31-202 Krakow, Poland; 4 Department of Radiology, Poznan University of Medical Sciences, 61-701 Poznan, Poland

**Keywords:** optical coherence tomography, intracoronary imaging, coronary artery aneurysm, coronary artery ectasia, atherosclerosis, coronary artery disease

## Abstract

**Background:** Coronary artery aneurysm and ectasia (CAAE) represent uncommon forms of coronary artery disease characterized by abnormal arterial dilatation and complex remodeling. The mechanisms underlying their development remain poorly defined. Optical coherence tomography (OCT) provides high-resolution evaluation of plaque morphology and vessel wall structure, offering insights into the pathophysiology of CAAE. **Methods:** We analyzed 21 patients with angiographically confirmed CAAE who underwent intracoronary OCT. Dilated segments were compared with adjacent non-dilated reference segments. Quantitative measurements included the maximal dilated segment’s diameter, reference diameter, and intima–media thickness. Qualitative assessment focused on plaque composition, calcification, neovascularization, fibrous cap characteristics, and thrombus. **Results:** Aneurysmal segments displayed larger lumen dimensions but no proportional increase in plaque burden, consistent with exaggerated positive remodeling. Compared with non-aneurysmal regions, CAAE segments exhibited significantly smaller calcification arcs and a lower prevalence of lipid plaques and neovascularization, suggesting a heterogeneous and potentially more fibrotic remodeling pattern. Classical features of plaque vulnerability were not consistently present in dilated segments, suggesting that hemodynamic factors, such as disturbed flow and stenosis, may contribute substantially to the thrombotic risk. **Conclusions:** OCT reveals distinct structural and compositional characteristics in CAAE, supporting the concept of maladaptive remodeling rather than uniformly unstable plaque. High-resolution intracoronary imaging enhances understanding of CAAE pathophysiology and may facilitate individualized clinical assessment and management.

## 1. Introduction

**Figure 1:**
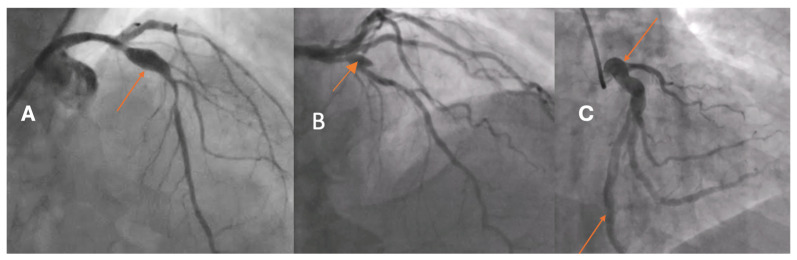
Morphological type of coronary artery dilatation. Coronary angiograms showing (**A**) AP cranial view of a fusiform aneurysm of the proximal segment of the left anterior descending artery (LAD) (arrow). (**B**) RAO cranial view of a saccular aneurysm on the bifurcation of LAD. (**C**) AP caudal view of coronary artery ectasia of the left circumflex artery (LCx). Classical angiographic projections were performed with individual modifications to achieve optimal visualization of the dilatation.

Coronary artery aneurysm and ectasia (CAAE) are uncommon abnormalities of the epicardial coronary arteries, reported in approximately 0.15–5.3% of patients undergoing coronary angiography [1,2,3,4,5]. Based on the extent of arterial involvement and the relationship between the transverse and longitudinal dimensions, two morphological types are distinguished: coronary artery ectasia (CAE), defined as a diffuse dilatation involving at least one-third of the vessel length, and coronary artery aneurysm (CAA), a focal dilatation that may be saccular or fusiform (Figure 1). In both CAE and CAA, the dilated segment exceeds the reference vessel diameter by ≥1.5-fold.

CAAE carries important clinical implications. Dilated segments are prone to abnormal turbulent flow and thrombosis, which can lead to distal embolization, acute coronary syndromes, and, less commonly, rupture. Patients may also experience recurrent ischemia due to microembolization or coexisting obstructive coronary disease.

The pathophysiology of CAAE is heterogeneous, reflecting both congenital and acquired processes [6]. In adults, atherosclerosis is the predominant cause, whereas Kawasaki disease represents the most frequent etiology in children. Additional contributors include systemic connective tissue disorders, infectious etiologies, vasculitides, congenital anomalies, genetic predisposition, and idiopathic cases [7]. Atherosclerosis is a complex, chronic inflammatory process that injures the arterial wall and drives maladaptive vascular remodeling. A central unanswered question is why only a small subset of individuals with atherosclerosis develop pathological dilatation of the coronary artery.

High-resolution intravascular imaging can help address this knowledge gap. Optical coherence tomography (OCT) offers the highest axial resolution among currently available intravascular modalities, enabling detailed assessment of plaque morphology and arterial wall microstructure within dilated and reference segments [8]. Although evidence to date is limited to small retrospective series and case reports, OCT and histopathology have suggested excessive positive remodeling with an altered intima-to-media thickness ratio, characterized by intimal thickening and medial thinning, together with destruction of musculoelastic elements, degradation of collagen and elastic fibers, and disruption of the elastic lamina [9,10,11]. The present study evaluates coronary artery remodeling in aneurysmal segments versus adjacent non-dilated segments using OCT, intending to identify distinct morphological features associated with CAAE.

## 2. Materials and Methods

The study population consisted of 21 patients with stable angina undergoing coronary angiography in whom CAA or CAE was identified. Patients were enrolled consecutively when CAAE was angiographically detected and when intracoronary OCT imaging of the dilated segment was technically feasible and clinically justified. Blood samples were obtained at the end of the index coronary angiography procedure.

We analyzed one dilated segment in each patient. All patients were evaluated using OCT during the index procedure. Qualitative OCT features were assessed using a binary classification (present/absent) for each analyzed segment. For every patient, the entire OCT pullback covering the aneurysmal/ectatic segment and the corresponding adjacent non-dilated reference segment was systematically reviewed. A qualitative feature was considered present if it was identified in any cross-section within the analyzed segment, in accordance with established OCT analysis standards used in prior intravascular imaging studies.

The assessment was not limited to a single frame. Still, it was performed across the full longitudinal extent of the segment of interest, ensuring comprehensive evaluation of plaque composition and vessel wall characteristics. This approach was chosen to account for the known heterogeneity of plaque morphology and remodeling patterns within both aneurysmal and non-aneurysmal coronary segments.

CAAE encompasses two phenotypes: CAE, defined as a diffuse dilatation of a coronary artery to ≥1.5 times the diameter of an adjacent normal reference segment that involves at least one-third of the vessel length, and CAA, defined as a focal dilatation of a coronary artery to ≥1.5 times the diameter of the nearest normal segment (Figure 1). CAA is morphologically classified as saccular when the transverse diameter exceeds the longitudinal dimension and as fusiform when the longitudinal dimension exceeds the transverse diameter with circumferential, spindle-shaped involvement of the vessel. A “giant” CAA is commonly designated when the diameter exceeds four times the reference caliber or is greater than 8 mm.

Regarding the classification of giant CAA, this definition was included for completeness and to align with established angiographic and clinical conventions. However, no giant aneurysms were identified in our cohort, and therefore, this distinction did not influence the analysis or conclusions. The size-based subclassification was not a determinant of our OCT comparisons.

**Figure 2:**
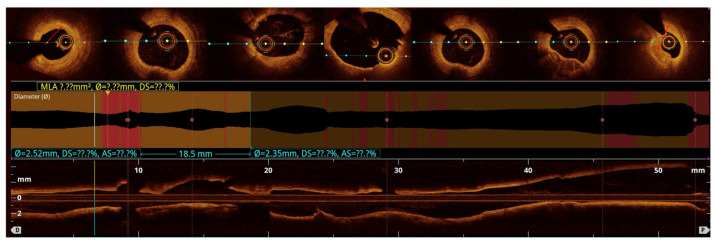
Coronary vessel lumen visible in OCT. Above, a transverse cross-section of the coronary vessel. In the middle and at the bottom, a longitudinal cross-section through the entire continuity of the vessel (distal -> proximal). Figure 2, Figure 3 and Figure 4: OCT images are displayed using the manufacturer-provided pseudo-color scale, where high signal intensity (strong backscattering) is represented by bright yellow/white, intermediate signal intensity by orange to red, and low signal intensity or signal-poor regions by dark brown to black. Signal attenuation results in progressive loss of signal intensity with depth.

OCT-based analysis of aneurysmal remodeling:

Quantitative analysis was performed using optical coherence tomography (OCT) pullbacks (The OPTIS Next Imaging System by Abbott) obtained during optimal contrast clearing and a stable catheter position (Figure 2). The aneurysmal segment was defined as the cross-section exhibiting the maximal luminal area within the visibly dilated portion of the vessel. The diameter was determined as the mean lumen and external elastic membrane (EEM) areas of proximal and distal reference cross-sections, located at least 5 mm away from the aneurysmal borders and appearing free or minimally affected by atherosclerosis, in accordance with established intravascular imaging standards [10,11]. When one or both adjacent segments were unsuitable due to diffuse disease, the least-diseased portion of the same vessel or the largest angiographically normal coronary artery was used as the reference [2,12]. CAA was defined as a focal luminal enlargement ≥1.5 times the reference diameter or >50% greater lumen area, consistent with prior angiographic and intravascular definitions [13].

**Figure 3:**
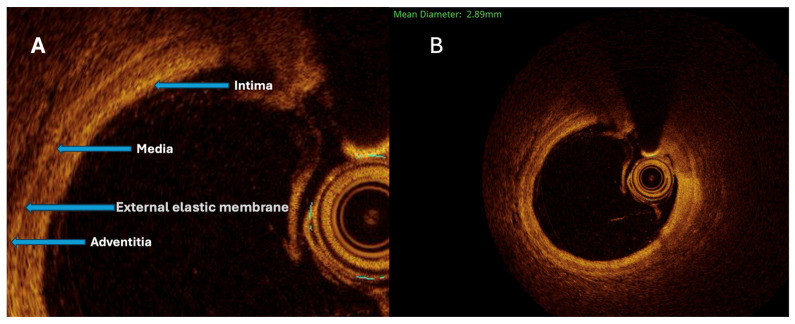
All three vessel layers (**A**) of the normal coronary vessel (**B**). The External Elastic Membrane (EEM) is between the hyporeflective media and the more heterogeneous adventitia.

The maximal aneurysm diameter (right, within the aneurysmal segment) is measured perpendicularly across the lumen at the point of most significant dilation. The reference diameter (left) is measured perpendicularly across the lumen of the adjacent, non-dilated (normal) segment of the same vessel, proximal to the aneurysm. The aneurysmal ratio is calculated as the maximal aneurysm diameter divided by the reference diameter; values ≥1.5 indicate aneurysmal remodeling according to standard interventional definitions. The normal coronary vessel, with all its layers, is presented in Figure 3.

**Figure 4:**
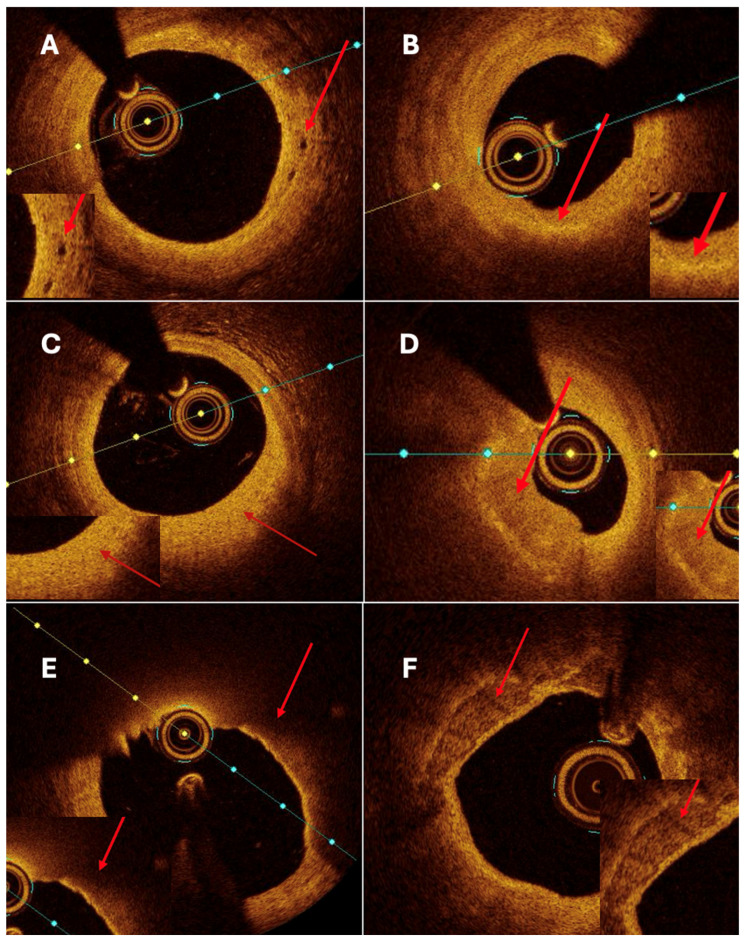
(**A**) neovascularization; (**B**) macrophages; (**C**) fibrotic plaque; (**D**) healed plaque; (**E**) lipid-rich plaque and TCFA; (**F**) calcified plaque. Abbreviations: TCFA, thin-cap fibroatheroma. Red arrows point at the particular pathology of interest.

Both quantitative and qualitative OCT analysis assessed vascular remodeling. Quantitative assessment included measurements of intimal and medial thickness, as well as the calculation of the intima-to-media thickness ratio. Qualitative evaluation was based on the identification of specific morphological features, including fibrosis, calcification, cellular infiltration, neovascularization, and thrombus formation. Homogeneous areas with strong backscattering and mild signal attenuation characterized fibrosis. Calcifications were defined as well-demarcated regions with low signal intensity and minimal attenuation. Cellular infiltration was identified as signal-rich bands with strong light attenuation resulting in shadowing. Neovascularization appeared as ellipsoidal, low-signal structures consistent with microvessels (Figure 4A). Thrombi were further categorized as white, defined by signal-rich, irregular masses with low attenuation, or red, characterized by high backscattering and strong attenuation.

Patients were eligible for inclusion if they presented with stable angina, had no significant coronary stenosis, and were diagnosed with CAAE in a vessel with a reference diameter between 2.0 and 3.5 mm, defined as a dilation at least 1.5 times greater than the diameter of the patient’s largest normal coronary artery. Written informed consent was obtained from all participants before their inclusion in the study. Exclusion criteria comprised CAAE located in peripheral vessels with a reference diameter ≤2.0 mm or other technical limitations preventing adequate OCT evaluation; presence of acute inflammatory disease (hsCRP > 100 mg/L); active malignancy; hemodynamic instability or cardiogenic shock; significant coagulation abnormalities; acute renal failure or chronic kidney disease with a glomerular filtration rate (GFR) <30 mL/min; known allergy to iodine-based contrast agents; or lack of informed consent.

Statistical analysis was performed using PQStat software (version 1.8.4; PQStat, Poznań, Poland; https://pqstat.pl). Descriptive statistics, including median, minimum, and maximum values, were calculated for continuous variables, and frequency distributions were generated for categorical data. Data normality was assessed using the Shapiro–Wilk test (α = 0.05). Comparisons between healthy vessel segments and aneurysmal or ectatic regions were conducted using the Mann–Whitney U test for non-normally distributed data and the independent samples Student’s *t*-test for normally distributed data. Categorical variables were compared using the Chi-square (χ^2^) test. A *p*-value < 0.05 was considered statistically significant. Due to the relatively small sample size, some comparisons did not reach statistical significance; therefore, observed patterns and trends in selected clinical parameters were also evaluated descriptively.

## 3. Results

Given the exploratory nature of the study and the limited sample size, effect sizes and relative percentage differences between aneurysmal/ectatic and non-dilated segments were additionally calculated to support the interpretation of observed trends. Effect sizes were reported using appropriate measures for continuous variables (e.g., median or mean differences with relative percentage change), alongside corresponding descriptive statistics. This approach was intended to provide complementary information on the magnitude and potential clinical relevance of differences that did not reach conventional thresholds for statistical significance.

The study population, presented in Table 1, consisted predominantly of men (71.4%), with a median age of 65 years (range, 36–82). Most patients were overweight, with a median BMI of 27.4 kg/m^2^, and over one-third (36.8%) met the criteria for obesity (BMI > 30 kg/m^2^). Arterial hypertension was the most common comorbidity, affecting 80.9% of the cohort, followed by hyperlipidemia (71.4%) and diabetes mellitus (38.1%), all of which are established cardiovascular risk factors. None of the patients had insulin-dependent diabetes.

A history of myocardial infarction was present in 42.9%, and 28.6% had undergone previous PCI, whereas none had undergone CABG. Heart failure was diagnosed in 19.0% of patients, with a median ejection fraction (EF) of 45% (30–60%). Other comorbidities included chronic kidney disease (14.3%), systemic connective tissue disease (14.3%), chronic obstructive pulmonary disease (9.5%), and peripheral artery disease (4.8%). One patient had a history of Kawasaki disease (4.8%), while no cases of Takayasu arteritis or other systemic vasculitides were observed.

Regarding pharmacotherapy, the vast majority received dual antiplatelet therapy (P2Y_12_ inhibitor and acetylsalicylic acid, both 90.5%) as well as beta-blockers (95.2%) and statins (95.2%). ACEI or ARB were used in 80.9% of patients, while 38.1% were on aldosterone antagonists and 19.0% on SGLT2 inhibitors.

Clinically, over half of the patients (57.1%) presented with ST-segment elevation myocardial infarction (STEMI), 38.1% with chronic coronary syndrome, and 4.8% with unstable angina. Laboratory parameters revealed normal median hemoglobin (14.8 g/dL), platelet count (226 × 10^3^/µL), and lipid profile (median total cholesterol 5.06 mmol/L, LDL-C 3.14 mmol/L, HDL-C 1.31 mmol/L, triglycerides 1.35 mmol/L).

The majority of aneurysmal or ectatic lesions were located in the left anterior descending artery (LAD) (42.9%), followed by the right coronary artery (RCA) (28.6%) and the left circumflex artery (LCx) (9.5%). Only one case (4.8%) involved the left main coronary artery (LMCA), while no aneurysms were identified in the diagonal, intermediate, or marginal branches. Baseline angiographic characteristic is presented in Table 2. Regarding morphology, CAE was the predominant form, observed in 61.9% of patients, whereas fusiform aneurysms accounted for 28.6% and saccular aneurysms for 9.5%. The median maximum lumen diameter was 5.3 mm (IQR 4.7–5.9), and the median lesion length was 10.0 mm (IQR 8.1–12.5). No giant aneurysms were detected in the study population.

These findings indicate that aneurysmal and ectatic changes most frequently affect the LAD and predominantly manifest as diffuse ectatic remodeling rather than discrete saccular dilatations, supporting the concept of progressive vessel wall weakening and outward remodeling along the major coronary branches.

OCT analysis revealed distinct morphological differences between aneurysmal and non-aneurysmal coronary segments. OCT analysis in aneurysmal and non-aneurysmal segments is presented in Table 3. The maximum calcification arc was significantly smaller in aneurysmal regions compared to non-CAAE segments (97.5° vs. 232.5°, *p* = 0.009). At the same time, fiber cap thickness did not show statistical significance in comparison between the two groups. Lipid plaques were significantly less frequent in CAAE segments (42.9% vs. 76.2%, *p* = 0.009), whereas neovascularization was utterly absent in the aneurysmal group but present in 38.1% of non-CAAE lesions (*p* = 0.002).

No significant differences were observed for intima thickness, media thickness, intima/media ratio, calcifications (Figure 4F), or nodules. Trends toward higher thrombus presence and macrophage infiltration were noted in non-CAAE segments, though they did not reach statistical significance.

Overall, aneurysmal segments exhibited thinner fibrous caps and smaller calcification arcs, suggesting a distinct remodeling pattern compared with non-aneurysmal coronary regions.

These findings indicate that aneurysmal vessel segments differ structurally and compositionally from non-aneurysmal coronary regions. Although intimal and medial thickness did not differ significantly between groups, aneurysmal segments demonstrated a trend toward a higher intima-to-media ratio, suggesting altered wall architecture. Importantly, CAAE regions showed significantly smaller calcification arcs and a lower prevalence of lipid plaques, together with an absence of neovascularization, compared with non-CAAE segments.

Although thin-cap fibroatheroma (TCFA) (Figure 4E) was more frequently identified in aneurysmal segments, fibrous cap thickness did not differ significantly between aneurysmal and non-aneurysmal regions. This apparent discrepancy likely reflects the composite nature of the TCFA definition, which incorporates plaque morphology, lipid distribution, and cap thickness in addition to cap thickness alone. In the context of coronary artery aneurysm and ectasia, TCFA may therefore represent a heterogeneous plaque phenotype within chronically remodeled vessel segments rather than a uniformly unstable lesion characterized by markedly thinned fibrous caps. These features suggest that CAAE is associated with qualitative alterations in plaque composition and vessel wall structure rather than an overall increase in plaque burden.

## 4. Limitations

We acknowledge that the inclusion of patients with different clinical presentations, including stable angina, chronic coronary syndrome, and STEMI, introduces a degree of clinical heterogeneity that may influence certain OCT-derived features, such as thrombus presence, macrophage infiltration, or fibrous cap appearance. However, our primary analysis focused on within-patient comparisons between aneurysmal (or ectatic) segments and adjacent non-dilated reference segments, thereby minimizing the confounding impact of systemic inflammatory or acute-phase effects related to clinical presentation. Moreover, OCT acquisition and analysis were performed using standardized criteria across all patients. We have now clarified this point in the Discussion and explicitly acknowledged clinical heterogeneity as a limitation, emphasizing that our findings should be interpreted in the context of mixed clinical presentations and warrant confirmation in larger, more homogeneous cohorts.

We agree that the higher prevalence of TCFA in aneurysmal segments should be interpreted with caution in the context of the overall plaque phenotype observed in our study. Although TCFA was more frequently identified in aneurysmal segments, this finding was not accompanied by a significant difference in fibrous cap thickness, nor by features typically associated with heightened plaque vulnerability, such as extensive lipid burden or neovascularization. In CAAE, TCFA may reflect altered plaque architecture within a chronically remodeled vessel wall rather than an unequivocally unstable phenotype. This interpretation is further tempered by the small sample size, which limits the ability to draw definitive conclusions regarding vulnerability patterns in aneurysmal disease.

A further limitation of the present study is the lack of a dedicated subgroup analysis comparing focal coronary artery aneurysms and diffuse coronary artery ectasia. Although these entities may differ in extent and clinical presentation; however, both are widely regarded as part of a shared spectrum of coronary artery aneurysmal and ectatic disease characterized by abnormal positive remodeling and dilatation. In the present study, the overall sample size was limited, reflecting the relative rarity of CAAE in routine clinical practice. Further subdivision into aneurysm and ectasia subgroups would therefore result in very small groups with insufficient statistical power and a high risk of spurious or misleading associations. For this reason, and in keeping with the exploratory nature of this study, we analyzed CAAE as a unified phenotype to focus on common structural and compositional remodeling patterns identified by OCT. We have clarified this rationale in the revised manuscript and acknowledge that larger, dedicated cohorts will be required to enable robust subgroup analyses in future studies.

We agree that, given the relatively small sample size and the number of quantitative and qualitative comparisons performed, there is an inherent risk of type I error. Accordingly, our findings are interpreted as exploratory and hypothesis-generating rather than confirmatory, and we emphasize the need for larger, prospective studies to validate these observations.

## 5. Discussion

In this study, intracoronary OCT was used to characterize vessel wall morphology and plaque composition in CAAE compared with adjacent non-dilated segments. Our findings demonstrate that aneurysmal regions exhibit a distinct remodeling phenotype, marked by outward (positive) enlargement of the vessel wall without a corresponding increase in plaque burden. Although lumen and external elastic membrane dimensions were larger in CAAE, overall plaque accumulation remained similar to that of reference segments, supporting the concept of exaggerated positive remodeling in these lesions. This pattern is consistent with previous observations that compensatory vascular enlargement can preserve lumen size despite the presence of underlying atherosclerosis.

Beyond these geometric changes, our OCT analysis reveals that CAAE is associated primarily with qualitative alterations in plaque architecture rather than increased plaque volume. Aneurysmal segments showed significantly smaller calcification arcs, reduced frequency of lipid plaques, and a complete absence of neovascularization. At the same time, thin-cap fibroatheroma (TCFA) was more prevalent in aneurysmal regions than in the non-aneurysmal areas. This constellation of features differs from classical vulnerability markers typically described in positively remodeled segments—such as large lipid cores and active neovascular networks—and instead suggests a form of chronic arterial wall injury characterized by structural weakening and altered plaque composition.

OCT-defined thin-cap fibroatheroma (TCFA) identified within dilated coronary segments may not reflect the same biological substrate or prognostic significance as TCFA in non-dilated coronary arteries. In the setting of coronary artery aneurysm and ectasia, altered vessel geometry, disturbed flow patterns, and chronic wall remodeling may influence the appearance of plaque and the assessment of the fibrous cap on OCT. We also note that these findings should be interpreted in the context of the small sample size and warrant confirmation in larger, dedicated studies integrating hemodynamic and clinical outcome data.

Taken together, these observations imply that CAAE may represent a heterogeneous and more advanced stage of maladaptive atherosclerotic remodeling, driven less by acute plaque instability and more by long-standing wall degeneration. This mechanistic profile may help explain the variable clinical behavior of CAAE and underscores the importance of individualized assessment integrating anatomical, compositional, and functional parameters. The pathophysiology of CAAE is complex and likely multifactorial. In adults, atherosclerosis is the predominant substrate, with chronic inflammation, matrix degradation, and destruction of musculoelastic elements contributing to loss of medial support and progressive outward dilatation.

Histopathological and intravascular imaging data indicate that the balance between intimal thickening and medial thinning, as well as the degradation of collagen and elastic fibers, is a key determinant of whether a lesion remodels concentrically, remains stable, or evolves into ectasia or an aneurysm [7]. Our OCT findings, which demonstrate altered intima–media relationships and distinct calcific and lipid patterns in dilated segments, are consistent with the concept of maladaptive remodeling.

Beyond atherosclerosis, OCT data from patients with KD provide important insight into shared pathways of coronary wall injury. Studies in the late convalescent phase of KD have consistently shown intimal thickening, medial disruption, and layered plaques suggestive of prior thrombosis, not only in persistent or regressed aneurysms but also in angiographically “normal” segments [14]. These observations underscore that once the arterial wall has been structurally damaged—whether by vasculitis in childhood or by atherosclerosis in adulthood—diffuse pathological remodeling can occur, extending beyond clinically evident aneurysmal sites. Although the etiology in our cohort was predominantly atherosclerotic, the parallels to KD support the notion that CAAE represents a final common pathway of chronic injury, inflammation, and matrix disorganization.

OCT has also been instrumental in elucidating the mechanisms of dilatation formation after coronary stent implantation. Case reports and small series have demonstrated that post-stent aneurysms may represent true aneurysms, characterized by the preservation of the three-layered arterial architecture and outward expansion, or pseudoaneurysms associated with focal wall disruption [11]. In these settings, OCT can distinguish neointimal proliferation, malapposition, tissue prolapse, and late-acquired wall defects, thereby clarifying the mechanism of stent-related aneurysm and guiding subsequent management (e.g., conservative therapy, covered stent, or surgical referral). Although stent-related aneurysms were not the primary focus of our cohort, these data illustrate the unique value of OCT for the mechanistic understanding of aneurysmal dilatations.

From a clinical perspective, intracoronary imaging complements angiography by revealing the underlying substrate of CAAE. Angiography alone cannot differentiate between thrombus-filled sacs, heavily calcified ectatic segments, thin-cap fibroatheroma, or predominantly fibrotic aneurysmal walls. OCT offers subcellular-level resolution, allowing precise identification of lipid-rich plaques, thin fibrous caps, macrophage accumulation, neovascularization, and thrombus [15]. Such information can refine risk stratification, influence antithrombotic strategies, and support decisions regarding revascularization, particularly when aneurysmal segments coexist with intermediate stenoses or when the culprit lesion is ambiguous.

Beyond imaging-based characterization, increasing attention has been directed toward therapeutic strategies aimed at modifying maladaptive vascular remodeling. Although coronary artery aneurysm and ectasia represent distinct clinical entities, emerging data from other vascular models provide critical mechanistic insights into potential approaches for stabilizing diseased vessel walls. In this context, a recent preclinical study demonstrated that a bismuth-infused perivascular wrap, enabling the local delivery of mesenchymal stem cells, significantly attenuated neointimal hyperplasia in rat arteriovenous fistulas, underscoring the role of targeted perivascular and adventitial modulation in vascular remodeling processes [13].

While the biological setting of arteriovenous fistulas differs from coronary aneurysmal disease, both conditions share key features of dysregulated vascular wall biology, including altered smooth muscle cell behavior, extracellular matrix remodeling, and chronic inflammatory signaling. Our OCT findings, which reveal qualitative plaque alterations and evidence of chronic arterial wall degeneration in aneurysmal segments, support the concept that coronary artery aneurysm and ectasia may similarly benefit from strategies aimed at restoring structural integrity rather than solely addressing luminal stenosis or plaque burden. Although speculative, localized biologic or biomaterial-based interventions targeting the vessel wall may represent a future therapeutic direction for mitigating progression or thrombotic risk in coronary aneurysmal disease. Further translational studies will be required to determine whether such approaches can be adapted safely and effectively to the coronary circulation [13].

Complementary evidence supporting this concept has emerged from additional preclinical studies employing perivascular therapeutic delivery platforms. In particular, controlled local release of pharmacologic agents such as rosuvastatin or rapamycin from electrospun, bismuth nanoparticle-infused perivascular wraps has been shown to promote favorable vascular remodeling and attenuate pathological neointimal hyperplasia in arteriovenous fistula models by modulating inflammation, smooth muscle cell proliferation, and extracellular matrix turnover. Although these experimental systems differ anatomically and hemodynamically from the coronary circulation, the underlying principle of targeted modulation of vessel wall biological pathways to influence remodeling outcomes is highly relevant to coronary artery aneurysms and ectasias. In CAAE, where medial weakening, matrix disorganization, and dysregulated smooth muscle behavior appear to play a central role in aneurysmal progression, such localized approaches may offer a conceptual framework for future therapeutic exploration. When combined with high-resolution intracoronary imaging, particularly OCT-based phenotyping of plaque composition and wall structure, these strategies could ultimately facilitate the identification of patient-specific targets for stabilizing aneurysmal segments and reducing the risk of thrombotic or ischemic events [14].

Our findings also highlight the limitations of extrapolating concepts of plaque vulnerability derived from non-dilated coronary segments to aneurysmal disease. Classical markers of vulnerability (large lipid cores, thin caps, and rich neovascularization) may not manifest in a uniform way in CAAE, where flow disturbances, low shear stress, and stasis-related thrombosis add hemodynamic triggers to the structural substrate [11]. The absence or relative paucity of neovascularization and extensive lipid pools in some aneurysmal segments in our study suggests that thrombotic risk in CAAE may be driven as much by abnormal flow and large luminal chambers as by plaque composition per se. This reinforces the need for individualized assessment that integrates anatomy, plaque features, and physiological or flow-based data.

Several limitations should be acknowledged. First, the sample size was small and derived from a single center, limiting the statistical power and generalizability of our observations. Second, OCT provides exquisite resolution but limited penetration depth; medial and adventitial changes may therefore be underestimated compared with IVUS or histopathology. Third, follow-up data were incomplete, and event rates were low, precluding robust correlations between OCT features and clinical outcomes. Larger, prospective studies integrating OCT, IVUS, and non-invasive imaging, together with standardized clinical follow-up, are needed to determine which combinations of remodeling patterns and plaque characteristics best predict adverse events in patients with CAAE.

Significantly, the prognostic relevance of aneurysmal and ectatic remodeling extends beyond structural characterization. Clinical data demonstrate that coronary artery ectasia is associated with a higher incidence of adverse cardiovascular events, particularly in the setting of acute coronary syndrome. In a recent study evaluating outcomes in ACS patients with CAE, ectasia was identified as a marker of increased thrombotic risk and recurrent ischemic complications, supporting the concept that dilated coronary segments represent a high-risk substrate rather than a benign anatomical variant. These observations align with our findings that aneurysmal segments possess unique morphological and hemodynamic features that may predispose to thrombosis independently of classical plaque vulnerability [15].

We should consider the potential interaction between intracoronary imaging and vascular medical devices in the context of coronary artery aneurysmal disease. As highlighted in the comprehensive review by Kawsara and colleagues, the interventional management of coronary artery aneurysms often involves the use of covered stents, coils, or other endovascular devices, particularly in cases with concomitant obstructive disease or a high thrombotic risk. However, the optimal strategy remains undefined due to the limited availability of outcome data [16,17].

With respect to OCT imaging, metallic components, such as stent struts or metallic nanoparticles, introduce specific imaging artifacts. Metallic surfaces typically produce high backscatter, accompanied by signal shadowing, which can obscure underlying tissue and limit the visualization of deeper vessel wall structures in the region immediately adjacent to the metal. It is a known limitation of OCT and other high-resolution intravascular modalities in the setting of metallic implants. In clinical practice, techniques such as frame selection, pullback optimization, and cross-modality correlation with intravascular ultrasound (IVUS) or angiography are often employed to mitigate these effects when assessing stented segments or devices. While the present study did not include cases with extensive metallic implants or nanoparticle delivery systems, future research integrating OCT with device-based therapies in CAAE will need to account for these imaging limitations. Prospective multimodality studies that evaluate OCT alongside IVUS or newer imaging methods may enhance the assessment of device-vessel interactions and aid in defining the utility of OCT in patients with coronary aneurysmal disease and vascular medical devices.

Taking everything into consideration, intravascular imaging modalities, particularly OCT, play a pivotal role in elucidating the mechanisms underlying acute coronary syndromes by enabling high-resolution assessment of plaque morphology, thrombus, and vessel wall pathology. Recent evidence suggests that OCT-guided evaluation enhances lesion characterization, risk stratification, and interventional decision-making beyond the use of angiography alone. These capabilities are especially relevant for atypical patterns of coronary disease, such as coronary artery aneurysm and ectasia, where detailed microstructural assessment may clarify mechanisms of remodeling and thrombosis that are not apparent on conventional imaging. The expanding role of intravascular imaging in ACS therefore supports its broader application in complex coronary phenotypes, including aneurysmal disease [18,19].

This clinical relevance is further illustrated by recent case-based evidence demonstrating the diagnostic challenges posed by coronary artery ectasia in acute coronary syndrome. In a reported case of ACS occurring in the setting of diffuse CAE, angiographic findings were initially misinterpreted as coronary perforation due to contrast stagnation and atypical dye appearance within the dilated vessel. The use of intravascular imaging enabled the accurate differentiation between true vessel injury and ectatic anatomy, thereby guiding appropriate percutaneous interventions and preventing unnecessary or inappropriate management. This example illustrates how altered flow dynamics and vessel geometry in CAE can complicate angiographic interpretation, underscoring the added value of high-resolution intravascular imaging for accurate diagnosis and informed procedural decision-making in complex coronary phenotypes [20].

Findings from the Coronary Artery Ectasia and Aneurysm Registry (CAESAR) further emphasize the clinical relevance of aneurysmal coronary disease. Registry data indicate that patients with coronary artery aneurysms and ectasia experience higher rates of adverse cardiovascular events and complications compared with individuals without dilated segments, highlighting that CAAE is not a benign anatomical variant, but a phenotype associated with distinct clinical outcomes. These real-world outcomes underscore the importance of detailed phenotyping and risk assessment in CAAE, complementing high-resolution imaging approaches such as OCT that can reveal microstructural and compositional differences not captured by angiography alone [21].

Despite these limitations, our study supports the role of OCT as a key invasive modality for evaluating coronary artery aneurysms and ectasia. By combining high-resolution assessment of plaque composition with quantitative analysis of remodeling, OCT improves our understanding of the pathophysiological substrate of CAAE and may contribute to more informed, patient-tailored management strategies.

## 6. Conclusions

- CAAE involves qualitative changes in plaque architecture rather than simply increased plaque burden, with aneurysmal segments showing distinct structural patterns.
- Traditional markers of plaque vulnerability do not consistently apply to aneurysmal disease, where hemodynamic disturbances contribute substantially to risk.
- Thrombosis in CAAE may be driven as much by abnormal flow conditions as by plaque composition, highlighting the need for multimodal evaluation.
- OCT provides valuable high-resolution insight into plaque morphology and remodeling patterns in CAAE, although its limited penetration and the small study cohort warrant caution.
- Further large-scale, multimodality studies are required to determine which structural and remodeling features best predict adverse outcomes in CAAE.

## Figures and Tables

**Table 1:** Baseline clinical characteristics.

Variable	Study Population (*n* = 21)
Age (years)	65 (36–82)
Male	15 (71.4)
BMI (kg/m^2^)	27.4 (20.3–33.6)
HA	17 (81)
DM	8 (38.1)
IDDM	0 (0)
Hyperlipidemia	15 (71.4)
HF	4 (19.1)
EF, %	45 (30–60)
Smoking	8 (38.1)
Obesity (BMI > 30)	7 (36.8)
CAD family history	6 (28.6)
Post MI	9 (42.9)
Post PCI	6 (28.6)
Post CABG	0 (0)
Stroke	0 (0)
COPD	2 (9.5)
PAD	1 (4.8)
AF	1 (4.8)
CKD (GFR < 60)	3 (14.3)
Systemic connective tissue disease	3 (14.3)
Kawasaki Disease	1 (4.8)
Takayasu disease	0 (0)
Other systemic vasculitis	0 (0)
History of active cancer	3 (14.3)
Laboratory results	
HGB (g/dL)	14.8 (10.3–18.7)
PLT (×10^3^/µL)	226 (109–480)
WBC (×10^3^/µL)	8.9 (4.7–18.0)
Total Cholesterol (mmol/L)	5.1 (2.8–6.4)
HDL-C (mmol/L)	1.3 (0.8–2.0)
LDL-C (mmol/L)	3.1 (1.0–5.2)
TG (mmol/L)	1.4 (0.4–2.8)
eGFR (mL/min/1.73m^2^)	80.8(≥90 mL/min/1.73 m^2^)
Creatinine (µmol/L)	90 (55.8–181)
hsCRP (mg/L)	3.24 (0.49–228)
Drugs on admission	
P2Y12i	19 (90.5)
ASA	19 (90.5)
Anticoagulants	5 (23.8)
B-blocker	20 (95.2)
Calcium-channel blocker	5 (23.8)
Statin	20 (95.2)
ACEI/ARB	17 (81.0)
Oral antidiabetic drugs	7 (33.3)
Insulin	0 (0)
Aldosterone antagonist	8 (38.1)
Sacubitril + valsartan	2 (9.5)
SGLT2 inhibitor	4 (19.1)
Nitrate	1 (4.8)
Clinical presentation	
STEMI	12 (57.1)
NSTEMI	0 (0)
Unstable angina	1 (4.8)
Chronic coronary syndrome	8 (38.1)

Continuous Variables are presented as median (interquartile range), and categorical variables as number (percentage). Abbreviations: HA, arterial hypertension; DM, diabetes mellitus; IDDM, insulin-dependent diabetes mellitus; HF, heart failure; EF, ejection fraction; CAD, coronary artery disease; MI, myocardial infarction; PCI, percutaneous coronary intervention; CABG, coronary artery bypass grafting; COPD, chronic obstructive pulmonary disease; PAD, peripheral artery disease; AF, atrial fibrillation; HGB, hemoglobin; PLT, platelet count; WBC, white blood cells; HDL-C, high-density lipoprotein cholesterol; LDL-C, low-density lipoprotein cholesterol; TG, triglycerides; ASA, acetylsalicylic acid; ACEI, angiotensin-converting-enzyme inhibitors; ARB, angiotensin receptor blockers; NSTEMI, non-ST-elevation myocardial infarction; STEMI, ST-elevation myocardial infarction.

**Table 2:** Baseline angiographic characteristic.

Variable	Study Population (*n* = 21)
CAAE localization	
LMCA	1 (4.8)
LAD	9 (42.9)
LCx	2 (9.5)
RCA	6 (28.6)
CAAE morphology	
CAA saccular	2 (9%)
CAA fusiform	6 (29%)
CAE	13 (62%)
Maximum lumen diameter, mm	5.3 (4.72–5.88)
Maximum length of CAA, mm	10 (8.1–12.5)
Giant aneurysm	0

Continuous Variables are presented as median (interquartile range), and categorical variables as number (percentage). Abbreviations: LMCA, left main coronary artery; LAD, left anterior descending artery; LCx, circumflex artery of the left anterior descending coronary; RCA, right coronary artery.

**Table 3:** Optical coherence tomography analysis in aneurysmal and non-aneurysmal segments.

	CAAE (*n* = 21)	Non-CAAE (*n* = 21)	*p*-Value
Intima thickness, mm	0.2 (0.1–0.3)	0.15 (0.1–0.1)	0.133
Media thickness, mm	0.11 (0.1–0.2)	0.12 (0.1–0.2)	1.000
Intima/media ratio	1.6 (1.2–2.9)	1.0 (0.8–1.7)	0.057
Calcifications	12 (57.1)	12 (57.1)	0.739
Maximum calcification thickness, mm	0.9 (0.7–1.1)	1.2 (1.0–1.3)	0.150
Maximum calcification arc, degrees	97.5 (60.9–152.2)	232.5 (180–318.7)	0.009
Calcified nodule	5 (23.8)	7 (33.3)	0.552
Lipid plaque	9 (42.9)	16 (76.2)	0.009
Maximum lipid arc, Degrees	170 (130–180)	254.5 (180–360)	0.056
Fiber Cap Thickness, μm	60 (60–100)	60 (44.5–83.7)	0.431
TCFA	57.14%	23.81%	0.003
Macrophages	6 (28.6)	12 (57.1)	0.073
Neovascularization	0	8 (38.1)	0.002
Thrombus	2 (9.5)	7 (33.3)	0.062

Continuous Variables are presented as median (interquartile range), and categorical variables as number (percentage). Abbreviations: TCFA, thin-cap fibroatheroma.

## Data Availability

The original contributions presented in this study are included in the article. Further inquiries can be directed to the corresponding author.
